# Loss of Speech after Chloroform

**Published:** 1871-01

**Authors:** 


Loss of Speech after Chloroform.—A servant girl, says the *Allg. Med. Centr. Zeit.*, for the sake of the extraction of a tooth, inhaled chloroform for a short time. On awaking, she had lost the power of speech, could not utter any sound whatever, and remained in that state for five weeks, in spite of various remedies, especially electricity. After this time she began to speak in a low tone, and was put under appropriate treatment. It is supposed that she suffered during anæsthesia from rupture of some cerebral vessel.—*Detroit Review of Medicine and Pharmacy.*

